# Olive Fruit and Leaf Wastes as Bioactive Ingredients for Cosmetics—A Preliminary Study

**DOI:** 10.3390/antiox10020245

**Published:** 2021-02-05

**Authors:** María de la Luz Cádiz-Gurrea, Diana Pinto, Cristina Delerue-Matos, Francisca Rodrigues

**Affiliations:** REQUIMTE/LAQV, Instituto Superior de Engenharia do Porto, Rua António Bernardino de Almeida, 4249-015 Porto, Portugal; diana.pinto@graq.isep.ipp.pt (D.P.); cmm@isep.ipp.pt (C.D.-M.)

**Keywords:** byproducts, olive leaves, olive fruits, revalorization, cosmetics, bioactive compounds, antioxidants

## Abstract

*Olea europaea* cultivar, native in the Mediterranean basin, has expanded worldwide, mainly due to the olive oil industry. This expansion is attributed to the benefits of olive oil consumption, since this product is rich in nutritional and bioactive compounds. However, the olive industry generates high amounts of wastes, which could be related to polluting effects on soil and water. To minimize the environmental impact, different strategies of revalorization have been proposed. In this sense, the aim of this work was to develop high cosmetic value added oleuropein-enriched extracts (O20 and O30), a bioactive compound from olive byproducts, performing a comprehensive characterization using high performance liquid chromatography coupled to mass spectrometry and evaluate their bioactivity by in vitro assays. A total of 49 compounds were detected, with oleuropein and its derivatives widely found in O30 extract, whereas iridoids were mainly detected in O20 extract. Moreover, 10 compounds were detected for the first time in olive leaves. Both extracts demonstrated strong antioxidant and antiradical activities, although O30 showed higher values. In addition, radical oxygen and nitrogen species scavenging and enzyme inhibition values were higher in O30, with the exception of HOCl and hyaluronidase inhibition assays. Regarding cell viability, olive byproduct extracts did not lead to a decrease in keratinocytes viability until 100 µg/mL. All data reported by the present study reflect the potential of industrial byproducts as cosmetic ingredients.

## 1. Introduction

Nowadays, olive trees (*Olea europaea* L.), native in the Mediterranean basin, have spread to many countries and adapted to different pedoclimatic conditions. This cultivar has expanded in Asia, America and Oceania mainly due to the olive oil industry, although the Mediterranean area is the most important region in terms of olives with almost 20 M tons of olive production in 2018 [1].

This globally expansion during the past two decades is mainly related to the health promoting properties attributed to olive oil consumption since this product contains monounsaturated fatty acids and bioactive compounds such as polyphenols, carotenoids or tocopherols, with pro healthy effects [2].

The world olive fruit production is 90% for olive oil and 10% for table olives [3]. For centuries, olive oil was used as major part of Mediterranean diet, for medicine, and as lamp fuel. Nowadays, olive oil is increasingly consumed for nutritional purposes and in the modern cosmetic industry. In any case, the olive oil industry generates high amounts of waste, particularly during the agricultural phase, i.e., harvesting tasks, and oil production. These byproducts are mostly olive pulp, pits, liquid wastes and leaves [4]. The disposal of these wastes is crucial because of their polluting effects on soil and water [5]. In this sense, over the years, composting has been the most popular technology for the revalorization of olive wastes producing fertilizers [6] or burning residues, although both these practices possess environmental risks. For this reason, recent research studies focus on new treatment and revalorization approaches that allow a sustainable recovery of valuable components from olive byproducts [7]. These valuable compounds present in herbal extracts have a long history as traditional remedies in different cultures due to their health benefits, such as antioxidant, anti-inflammatory, antiaging and antimicrobial, which are related to chronic disorders [8]. Therefore, there is a growing interest to reutilize these bioactive phytochemicals in various industrial sectors, such as food, pharmaceutical and cosmetic [2,4,9].

Among these byproducts, leaves, which represent about 5% of the weight of olives, have been used in folk medicine as remedies for treating diabetes or cardiovascular disorders [10]. Moreover, the scientific community has recently reported the beneficial health effects in humans [11], such as an exploratory randomized controlled trial for testing the beneficial effect of olive leaf tea that showed a significantly decrease in fasting plasma glucose levels [12]. However, further studies are necessary to clarify the candidate compounds responsible for these effects. In this sense, olive leaf composition has been studied by different authors [13,14,15,16,17], reporting the richness in bioactive compounds belonging to phenolic compounds, such as hydroxytyrosol, catechin, rutin, verbascoside, luteolin and oleuropein [18]. These compounds have been associated with antioxidant, anti-inflammatory, antiobesity or chemopreventive effects [15,19,20,21,22,23,24], among other activities. Specifically, oleuropein, which is formed by elenolic acid, glucose and hydroxytyrosol, acts as a powerful antioxidant at skin level [2]. The aim of this work was to develop powerful ingredients from olive byproducts rich in oleuropein through a combination of generally recognized as safe solvents (water/ethanol) and a feasible and sustainable scaling-up process for cosmetic applications. To this end, industrial olive byproduct extracts were analyzed by high-performance liquid chromatography (HPLC) coupled to quadrupole time of flight (QTOF) mass spectrometry (MS), and their bioactive potential was evaluated through antioxidant and reactive oxygen/nitrogen species scavenging assays as well as enzyme inhibition activities and cell viability effects. This paper proposes a first screening of the possibility of using bioactive compounds from olive fruit and leaves as effective antioxidants with interesting skin health benefits as well as a first barrier against reactive oxygen and nitrogen species related to aging, appraising their potential use for cosmetic purposes. In addition, the skin cell effects of olive byproduct extracts were assessed as an uttermost important preliminary analysis for cosmetics in order to determine the concentrations which show absence of toxicity in keratinocytes.

## 2. Materials and Methods

### 2.1. Chemicals and Reagents

All chemicals were of HPLC-MS grade and used as received. Acetic and formic acid and methanol for HPLC were purchased from Fluka (Sigma-Aldrich, Steinheim, Germany) and Lab-Scan (Gliwice, Sowinskiego, Poland), respectively. For solutions, ultrapure water was obtained with a Milli-Q system Millipore (Bedford, MA, USA), and absolute ethanol was purchased from VWR chemicals (Radnor, PA, USA).

To measure the Total Phenolic Content (TPC) and the antioxidant capacity, the following reagents were provided from the indicated suppliers: AAPH (2,2′-azobis-2-methyl-propanimidamide, dihydrochloride), ABTS [2,2′-azinobis (3-ethylbenzothiazoline-6-sulphonate)], ferric sulfate, fluorescein, Folin–Ciocalteu reagent, potassium persulfate, TPTZ (2,4,6-tripyridyl-S-triazine) and Trolox (6-hydroxy-2,5,7,8-tetramethylchroman-2-carboxylic acid) from Sigma-Aldrich (St. Louis, MO, USA). From Panreac (Barcelona, Spain), gallic acid, dehydrated sodium phosphate, trihydrated sodium acetate, sodium acetate, ferric chloride, hydrochloric acid and sodium carbonate were purchased. To measure Reactive Oxygen/Nitrogen Species (ROS/RNS) scavenging, dihydrorhodamine 123 (DHR), sodium hypochlorite solution with 4% available chlorine, β-nicotinamide adenine dinucleotide (NADH), phenazine methosulphate (PMS) and nitroblue tetrazolium chloride (NBT) were purchase from Sigma Aldrich (Steinheim, Germany). Dimethylsulfoxide (DMSO) was obtained from AppliChem (Darmstadt, Germany).

To measure hyaluronidase and elastase inhibitions, all reagents, hyaluronidase from bovine testes Type I–S, hyaluronic acid as substrate, sodium acetate/phosphate/chloride, bovine serum albumin, buffer HEPES, NaCl, human leucocyte elastase, substrate MeOSuc-Ala-Ala-Pro-Val-pNA and elastatinal, were purchase from Sigma (St. Louis, MO, USA).

For cell viability, human immortalized non-tumorigenic keratinocytes cell line HaCaT (ethnicity, Caucasian; age, 62 years; gender, male; tissue, skin) was obtained from CLS Cell Lines Service, Germany. Dulbecco’s Modified Eagle Medium (DMEM), Fetal Bovine Serum (FBS), GlutaMAX^TM^, Hank’s Balanced Salt Solution (HBSS), non-essential amino acids, penicillin, streptomycin and trypsin–EDTA were obtained from Invitrogen Corporation (Life Technologies, S.A., Madrid, Spain). Trypan blue dye was purchased from Gibco (Thermo Fisher Scientific, Waltham, MA USA). Tissue culture flasks were acquired from Orange Scientific (Braine-l’Alleud, Belgium).

### 2.2. Sample Preparation

Two different oleuropein-enriched extracts from *O. europaea* fruits and leaves (O20 = 20% oleuropein and O30 = 30% oleuropein + 10% triterpenes) were obtained from Natac company (Alcorcón, Madrid, Spain). These extracts were obtained by different process. Briefly, O20 was attained by a first ethanol extraction followed by filtration and concentration steps. After that, a second water extraction was made, followed by filtration, thermal treatment, vacuum drying, milling and standardization steps. O30 was obtained through two extraction processes running in parallel with different filtration and drying methodologies. The two milled final extracts were homogenized and standardized. The commercial extracts were supplied as dry residue then filtered with a 0.2 µm sterile filter, evaporated and lyophilized according to the manufacturer’s procedure. Both extracts (O20 and O30) were dissolved in ethanol:water (20:80; *v*:*v*) at a final concentration of 1 mg/mL for further experiments, except for the HPLC analysis (extracts were analyzed at a final concentration of 5 mg/mL) and cell assays (extracts at 1 mg/mL were dissolved in the culture medium used); then, extracts were vortexed for 1 min, sonicated for 20 min, centrifuged for 5 min at 17,000× *g* in a Sorvall ST 16 R centrifuge (Thermo Scientific, Leicestershire, UK), filtered through a 0.20 mm filter and kept at −20 °C under light-free conditions until further experiments. Preliminary studies were performed for each assay to investigate the influence of the solvents (water, ethanol and culture medium) in the composition of the extracts.

### 2.3. Chromatographic Conditions and Mass Spectrometry Detection

The ACQUITY UPLC H-Class System (Waters Corp., Milford, MA, USA) was used for the separation of the bioactive compounds from both extracts (5 mg/mL) at 22 °C with a Zorbax Eclipse Plus C18 column (1.8 μm, 150 × 4.6 mm). The mobile phases were acetic acid 0.5% (solvent A) and methanol (solvent B). This multistep linear gradient was applied: 0 min, 100% A; 5 min, 75% A; 20 min 61% A; 30 min, 40% A; 38 min, 0% A; 46 min, 100% A. The initial conditions were maintained for 10 min. The injection volume was 10 μL. The flow rate used was set at 0.4 mL/min.

The HPLC analysis was coupled to QTOF/MS (Synapt G2, Waters Corp., Milford, MA, USA). The determination of the compounds was carried out using an electrospray source operating in negative ionization mode under the following conditions: MS acquisition was performed using two parallel scan functions by rapid switching, in which one scan was operated at low collision energy in the gas cell (4 eV) and the other at an elevated collision energy (MSE energy linear ramp: from 20 to 60 eV); desolvation gas flow = 700 L h^−1^, desolvation temperature = 500 °C, cone gas flow = 50 L h^−1^, source temperature = 100 °C, capillary voltage = 2.2 kV, cone voltage = 30 V and collision energy = 20 eV. Full-scan mode was used (*m/z* = 50–1200). Scan duration was 0.1 s, and resolution was 20,000 FWHM. The MS data were processed through the open-source software MZmine.

### 2.4. Total Phenolic Content and Antioxidant Capacity Assays

The Folin-Ciocalteu method was carried out to measure the total phenolic content (TPC) as firstly validated by Singleton and Rossi [25], with minor modifications. This assay was directly performed in 96-well polystyrene microplates. Briefly, 30 μL of extracts (125 μg/mL) was mixed with 150 μL of Folin-Ciocalteu’s reagent (1:10, *v*/*v*) and 120 μL of 7.5% (*w*/*v*) Na_2_CO_3_ solution. The microplate was incubated at 45 °C for 15 min in a Synergy Mx Monochromator-Based Multimode Microplate reader (Bio-Tek Instruments Inc., Winooski, VT, USA) and then left to stand in the dark for 30 min, at room temperature. Then, the absorbance was measured at 765 nm, and TPC was calculated based on the calibration curves of gallic acid and expressed as mg of gallic acid equivalents (GAE)/g of dry extract. FRAP assay was performed according to the procedure described by Benzie and Strain [26], with slight modifications. An aliquot (40 μL) of extracts (250 μg/mL) was added to a 96-well microplate, along with 250 μL of FRAP reagent (composed by 10 parts of 300 mM sodium acetate buffer at pH 3.6, 1 part of 10 mM TPTZ solution and 1 part of 20 mM FeCl_3_ 6H_2_O solution). Ferrous sulphate was employed as standard (12.5–200 μM). After incubating the microplate at 37 °C for 10 min, the absorbance was recorded at 593 nm for 4 min. Results were expressed as mmol of ferrous sulphate equivalents (FeSO_4_)/g dry extract. The TEAC was determined by ABTS radical scavenging capacity assay following the procedure described by Miller et al. [27] with minor alterations. ABTS radical cation (ABTS^•+^) working solution was prepared by adding ABTS^•+^ stock solution to 2.45 mM potassium persulfate, and the final mixture was kept in the dark at room temperature, for 12 to 24 h. Prior to use, the ABTS^•+^ working solution was diluted with ultrapure water until reaching an absorbance of 0.700 (±0.02) at 734 nm. In a nutshell, 300 μL of ABTS^•+^ solution and 30 μL of extracts (250 μg/mL) or standard were mixed for 45 s, and the absorbance was measured after 5 min at 734 nm. Trolox (0.5–30 μM) was used as standard to plot a calibration curve. TEAC results were expressed as mmol of Trolox equivalents/g dry extract. The ORAC assay was performed to assess the capacity of the extracts to scavenge peroxyl radicals following the methodology described by Ou et al. [28] and modified by Laporta et al. [29]. A 56 nM fluorescein solution was prepared and kept for at least 30 min at 37 °C before use. Briefly, the reaction mixture (final volume of 210 μL) contained 40 nM fluorescein, 133 mM 2,2′-azobis-(2-methylpropionamine)-dihydrochloride (AAPH) and the tested concentrations of extracts (125 μg/mL) or Trolox (0.5–15 μM). Trolox was employed as standard to draw the calibration curve. All solutions were diluted in buffer solution consisting of 0.2 M NaH_2_PO_4_ and 0.2 M Na_2_HPO_4_ (20:80, *v*/*v*) at pH 7.4. The microplate was incubated at 37 °C, and the fluorescence was read at 520 nm (excitation wavelength at 485 nm) for 2 h. The results were expressed as mmol of Trolox equivalents/g dry extract. Three independent experiments were carried out for each assay.

### 2.5. Reactive Oxygen/Nitrogen Species Scavenging

The extracts and positive controls (gallic acid and catechin) were previously dissolved in phosphate buffer used for each procedure. These methods were performed in a Synergy HT Microplate Reader (BioTek Instruments, Inc., Winooski, VT, USA). Three independent experiments using six concentrations, in duplicate, were carried out for each assay. Results were calculated from the curves of inhibition percentage versus antioxidant concentration using the GraphPad Prism 7 software (GraphPad, La Jolla, CA, USA). Prior to the assays, the absorption of the extract was studied at the proper wavelengths for each method. The methods for superoxide radical scavenging activity, hypochlorous acid scavenging assay and nitric oxide scavenging assay were carried out following the reported literature [30]. Results were expressed as the inhibition in IC_50_. The IC_50_ values were automatically calculated by GraphPad Prism 7 software.

### 2.6. Enzyme Inhibitions

Hyaluronidase and elastase inhibitory assays were performed following the method describe by Nema et al., 2011 and 2013 [31,32]. Briefly, for hyaluronidase, the assay medium consisting of hyaluronidase (1.50 U) in 100 mL, 20 mM sodium phosphate buffer (pH 7.0) with 77 mM sodium chloride solution and 0.01% bovine serum albumin (BSA) was pre-incubated with 5 mL of the extracts for 10 min at 37 °C. Then, the assay was started by adding hyaluronic acid to the mixture. This was incubated for 45 min at 37 °C. The undigested hyaluronic acid was precipitated, and finally, the absorbance was measured at 600 nm. For elastase, the amount of released *p*-nitroaniline, which was hydrolyzed from the substrate, MeOSuc-Ala-Ala-Pro-Val-pNa, by elastase, was read with a maximum absorbance at 405 nm. In brief, MeOSuc-Ala-Ala-Pro-Val-pNa was prepared in buffer (pH 8.0), and this solution was added to the samples. The solutions were vortexed and pre-incubated at 37 °C for 10 min before an elastase solution was added. After that, they were incubated at 37 °C for 30 min, and the absorbance was measured at 405 nm. All measurements were made in triplicate.

### 2.7. Cell Viability

The cell viability assay was carried out according to the method described by Lameirão et al. [33]. HaCaT cells were individually maintained in Dulbecco’s modified Eagle’s medium (DMEM) with GlutaMAX^TM^-I, 10% inactivated fetal calf serum, 100 U/mL penicillin, 100 mG/mL streptomycin and 0.25 mG/mL amphotericin B, in a 5% CO_2_ environment at 37 °C (CellCulture^®^ CO_2_ Incubator, ESCO GB Ltd., UK). The number of viable cells was periodically assessed by the trypan blue exclusion assay. Keratinocytes (HaCaT cell line; passage 18–21) were exposed to different concentrations (0.1–1000 µg/mL) of extract for 24 h at 37 °C and in a water saturated atmosphere with 5% CO_2_ using an incubator. Following the supplier instructions, the 3-(4,5-dimethylthiazol-2-yl)-5-(3-carboxymethoxyphenyl)-2-(4-sulfophenyl)-2H-tetrazolium (MTT) assay was performed to estimate the intestinal cells’ viability by the determination of the number of viable cells through a colorimetric reaction. Briefly, cells were straightly placed in 96-well microplate (density of 25 × 10^3^ cells/well) and incubated at 37 °C for 24 h. Afterwards, cells were exposed to different concentrations of O20 and O30 extracts, positive control (1% (*v*/*v*) Triton X-100) and negative control (DMEM) for 24 h. After the supernatant removal, the MTT was added to each well, and the microplate was incubated at 37 °C for 3 h to promote the development of formazan crystals. Then, the MTT solution was removed, and the blue formazan crystals were eluted with DMSO. Absorbance was read at 570 nm with a background subtraction at 690 nm.

### 2.8. Statistical Analysis

All experiments were carried out at least in triplicate. The results were presented as mean ± standard deviation of at least three independent experiments. IBM SPSS Statistics 24.0 software (SPSS Inc., Chicago, IL, USA) was used for the statistical analyses of data. The differences between samples were investigated by one-way ANOVA, and *post hoc* comparisons of the means were performed with Tukey’s HSD test. In all cases, a denoting significance was accepted for *p* < 0.05. Regarding ROS and RNS scavenging assays, at least three independent experiments using six concentrations, in duplicate, were performed. Results were determined from the curves of inhibition percentage *versus* antioxidant concentration using GraphPad Prism 7 software (GraphPad, La Jolla, CA, USA).

## 3. Results and Discussion

### 3.1. Phenolic Profile of Olive Byproduct Extracts by HPLC-QTOF

**Figure 1:**
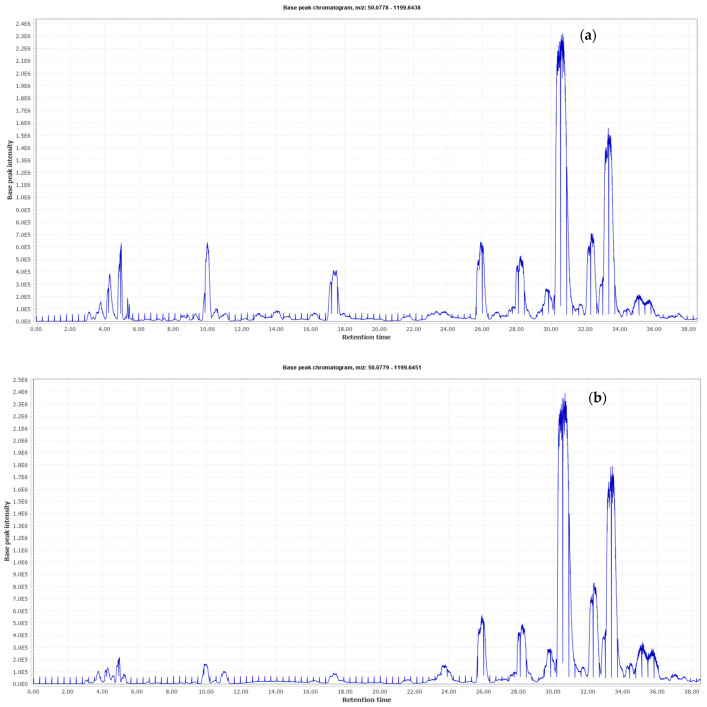
Base peak chromatograms of O20 (**a**) and O30 (**b**) olive byproduct extracts obtained by high performance liquid chromatography coupled to mass spectrometry.

Since it is important to have a better interpretation of the diversity of available phytochemicals contained in the bioactive ingredients from industrial olive byproducts, both O20 and O30 extracts were comprehensively characterized by HPLC-QTOF in order to analyze the polar bioactive fraction. The analytical platform provided two base peak chromatograms (BPC) that are shown in Figure 1. The characterized compounds are summarized in Table 1, numbered according to their elution order. This table includes retention times, proposed compound, *m/z*, molecular formula, MS/MS fragments and the presence of the compound in each extract. The identification of each compound was carried out by interpreting the accurate mass spectra information provided by MS and MS/MS and information previously reported in the literature. In this sense, a total of 49 compounds were detected, among which 28 were found in both extracts. This fact could be due to the differences in the extract production or even to the detection limits in the chromatographic analysis above all when the extracts are enriched in some specific compounds.

All compounds were classified into four major categories, oleuropein and its related compounds, iridoids, flavonoids and other compounds.

#### 3.1.1. Oleuropein and Its Related Compounds

It is known that the most abundant group of compounds in olive leaves is secoiridoids, mainly oleuropein (**40**, **42**, **44**, **45**) and its derivatives, but also the simple phenol hydroxytyrosol (**13**), a precursor of oleuropein, and verbascoside (**32**), a conjugated glucoside of hydroxytyrosol and caffeic acid [34]. It is worth noting that both olive byproduct extracts (O20 and O30) were attained with the purpose of developing oleuropein-enriched ingredients due to its reported antioxidant, anti-inflammatory, antimicrobial or antiaging effects [35]. For this reason, compounds 40, 42 and 44 (oleuropein isomers 1, 2 and 3) are the most abundant peaks. The presence of these isomers is due to the oleuropein isomer present that may have a glycosylation position distinct from hydroxytyrosol. Similarly to oleuropein, oleuropein derivatives were also detected, such as different isomers of hydroxyoleuropein (**29**, **34**), oleuropein glucoside (**31**, **38**) and oleuropein aglycone (**46**, **47**, **49**). Other derivatives, such as methoxyoleuropein (**39**) and another new related compound (**48**), were also identified. The last one detected at *m/z* 763 and molecular formula C_36_H_44_O_18_, which gave an MS/MS spectrum with main fragment ions at *m/z* 539 and 307, corresponded to oleuropein and its major product ion, respectively. To the best of our knowledge, this is the first time that this derivative has been reported, although a similar compound was previously described at *m/z* 864 by Cardoso et al. [36]. In addition, among secoiridoids, ligstroside (**43**) was also detected in both extracts as well as two isomers of elenolic acid glucoside (**18**, **19**). In a nutshell, tyrosol, hydroxytyrosol and oleuropein have been suggested as possible cosmetic ingredients due to their antioxidant and anti-inflammatory properties and claims of antiaging and hydration effects [37].

#### 3.1.2. Iridoids

This group comprises a wide number of monoterpenes and glucoside derivatives, whose structure may be derived from iridane. In addition, iridoids are the precursors of secoiridoids by opening the pentacyclic ring [38]. In the analyzed extracts, eight compounds were detected; among them, only three iridoids were found in both extracts corresponding to loganic acid (**10**); aralidioside (**12**), which has been reported in woody perennials for showing cardioprotective effects [39]; and acetylbarlein (**21**). These last two iridoids have been described for the first time in olive leaves in this work.

**Figure 2:**
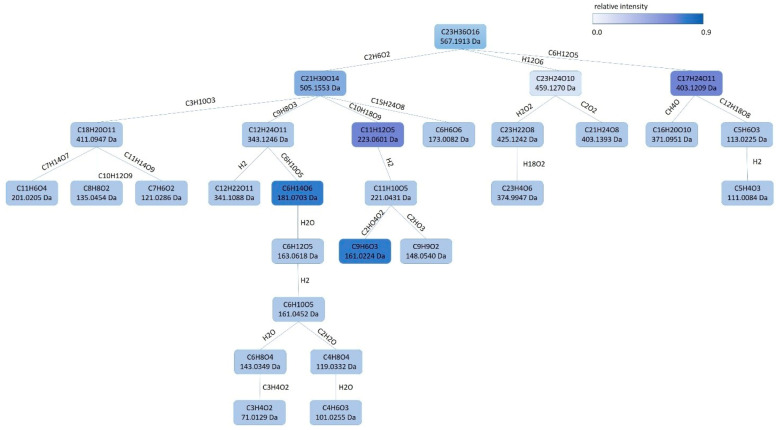
Fragmentation pattern tree from Sirius [43] of eribioside (**17**) found in O20 olive byproduct extract with shared MS ion spectrum with allobetonicoside (**16**) and another related compound (**15**).

Moreover, a group of iridoid glucoside derivatives was only found in O20 extracts. These compounds were tentatively identified for the first time in olive leaves as eriobioside (**17**), allobetonicoside (**16**) and other related compound (**15**) with a shared fragmentation pattern at *m/z* 181 and 161 as main fragments (Figure 2). Loganin (**20**) was also only detected in O20. However, a secologanol derivative from *Gentiana depressa* [40] was distinguished at *m/z* 687 and tentatively identified as depressine (**25**).

In conclusion, iridoids are interesting bioactive compounds largely used in skin disorders mostly due to their anti-inflammatory and antioxidant properties [41,42].

#### 3.1.3. Flavonoids

Flavonoids are among the main components of olive leaves presenting several biological properties [21]. Six compounds belonging to a flavonoid class were tentatively identified in O20 and O30 extracts, namely, taxifolin (**14**); paniculin (**26**), an isoflavone substituted by hydroxy groups that has been firstly reported in this work; two flavonols (kaempferol diglucoside (**27**) and rutinose (**36**)); and two flavones that were luteolin glucoside isomers (**37** and **41**).

#### 3.1.4. Other Compounds

Among this heterogeneous group, it was possible to detect organic acids, such as gluconic (**1**) and citric (**3**) acid, and different sugars (**2**, **5**, **8**). Moreover, simple phenols, namely, vanillin (**4**), which is commonly identified in olive leaves [44]; methylgallate glucoside (**6**); and the phenolic glycoside leunoriside A (**7**) were also characterized. The last one, leunoriside A, was reported as a stronger antioxidant than 3-tert-butyl-4-hydroxyanisole in *Leonurus japonicus* [45] and in the peel of chufa (*Eleocharis dulcis*) [46]. In addition, three isomers of oleoside (**9**, **11** and **22**) were detected at *m/z* 389. These compounds are present in a high amount in olive flowers [14].

On the other hand, cinnamoside (**24**), a terpene glycoside that was described in *Robusta coffee* [47], was also detected in O20 and O30 extracts. Moreover, three *o*-glycosyl compounds were detected at *m/z* 401 (**23**) and 415 (**28**, **30**). Among these primeveroside derivatives, **28** and **30** corresponding to phenetyl primeveroside isomers, these compounds have been previously found in olive leaves and reported for their capacity to inhibit AMP-activated protein kinase (AMPK) activity, an important regulator of cellular energy homeostasis, in a hypertrophic adipocyte model [23]. Finally, lignan syringaresinol (**33**) and the phenylpropanoid calceolarioside A (**35**) were also identified, the latter having been previously reported in olive leaves, although with other mass spectra data [23]. In this sense, this compound has also been identified in *Globularia orientalis* [48] with the same *m/z*, molecular formula and MS/MS spectrum that were obtained in O30 extract.

Overall, olive byproducts are excellent sources of bioactive compounds, particularly oleuropein and its derivatives, iridoids and flavonoids, with promising application in the cosmetic field due to interesting biological activities [49]. The valorization of olive wastes for the cosmetic industry will allow us to (i) recover bioactive compounds and use them to design high added value products with skin health effects; (ii) generate an additional economic resource for agro-industries; and (iii) decrease the environmental impacts of these agro-wastes [37].

### 3.2. Cosmetic Potential of Industrial Olive Byproduct-Enriched Extracts

#### 3.2.1. Total Phenolic Content and Antioxidant Activity

The antioxidant properties of phenolic compounds from plant sources have been widely reported in the literature [13,14,16,34]. Polyphenols are plant secondary metabolites that act as antioxidants and UV blockers, providing remarkable skin health effects and attenuating the effects of skin aging through the mitigation of the biochemical consequences of oxidation [2,37]. Antioxidants are innovative ingredients for skin care formulations due to their antioxidant and antimicrobial activities, protective effects from UV-mediated damages and inhibition of dermal proteinases [2,37]. Several plant-derived extracts have been employed in cosmetic products already marketed on a large scale [37]. The most common approaches to investigating the content and the antioxidant and antiradical potentials of phenolic compounds are by different in vitro methodologies, such as Folin-Ciocalteu and FRAP, TEAC or ORAC assays, respectively [50]. In this sense, Table 2 shows the obtained value for both olive byproduct extracts of TPC and antioxidant/antiradical activity assays. According to the obtained data, there are no significant differences in the *t*-test, but O30 achieved the highest values in all experiments. This fact could be related to the high content of oleuropein, which has been reported to be a potent antioxidant compound [35].

It is important to remark that both extracts demonstrated higher TPC and stronger antioxidant potential than other extracts previously reported in the literature [34,51,52,53]. The TPC of O20 and O30 extracts was 193 and 217 mg GAE/g dry extract. For example, olive leaf extracts from different cultivars were screened by Özcan et al. to evaluate their TPC, achieving values from 73.05 to 144.19 mg GAE/g dry extract, which are lower than those obtained in the present study [34]. Similarly, olive leaf extracts from Greek cultivars demonstrated lower TPC values by Folin-Ciocalteu methodology for all studied solvents (from 0.47 to 24 mg gallic acid/g dry extract) [54]. Khounani et al. also reported lower TPC for olive leaf methanolic extract (92.20 mg GAE/g dry extract) [55]. In addition, although optimized conditions of ultrasound assisted extraction of bioactive compounds from olive leaves were also evaluated in Greek cultivars, the best TPC value was attained at 37.44 mg gallic acid/g dry extract [51], considerably lower than the present results. Comparing to olive pomace from different cultivars (5.37–9.26 g GAE/kg dry extract), the olive by-product extracts analyzed in this study also had a higher TPC [56].

Regarding antioxidant capacity, both extracts showed higher values than those reported in literature [52,53]. The TEAC values were, respectively, 0.80 and 0.95 mmol eq. Trolox/g dry extract for O20 and O30 extracts. For example, a commercial micronized powdered olive leaf extract provided by Folhas de Oliva^®^ (Brazil) was used to evaluate in vitro antiradical action by TEAC assay, giving 0.592 mmol eq. Trolox/g dry extract [52,53]. Lower TEAC results were obtained for aqueous and 70% ethanol extracts from olive leaves (300–700 µmol eq. Trolox/g dry extract) [57]. Other genotypes from Turkey displayed similar ABTS^•+^ scavenging activity to that reported in Table 2, presenting a strong correlation between TEAC values and oleuropein content [53]. The results of ORAC assay were 3.91 and 3.99 mmol eq. Trolox/g dry extract. Additionally, Bermúdez-Oria et al. extracted pectin polysaccharides from olive pomace by hydrothermal treatment using different extraction temperatures (80–160 °C) [58]. Lower outcomes were reported for all extracts (50–400 µmol eq. Trolox/g dry extract) compared to the present study. Regarding FRAP assay, the results were 1.66 and 1.90 mmol eq. FeSO_4_/g dry extract for O20 and O30 extracts, respectively. Nunes et al. reported minor ferric reducing antioxidant power for olive pomace aqueous extract (66.38–101.51 g FeSO_4_/kg dry extract) compared to the present study [56]. All these results support the potential of O20 and O30 extracts as antioxidant sources for cosmetic applications.

#### 3.2.2. Reactive Oxygen/Nitrogen Species Scavenging

The production of reactive species provides beneficial health effects in various physiological processes, including host defense against infectious agents and cell signaling [59,60]. On the other hand, an imbalance between the pro-oxidant reactive species and the antioxidant defense capacity of cells promotes oxidative stress with harmful effects on cellular components (e.g., lipids, proteins and DNA) [59,61]. The overproduction of reactive oxygen species (ROS) and reactive nitrogen species (RNS) is closely related to pathophysiology of several diseases, such as cancer, diabetes, cardiovascular and neurological disorders as well as skin aging [59,60,61]. In this sense, plant extracts exert a protective role against oxidative stress-mediated conditions, helping to ensure the equilibrium of antioxidant defenses and to scavenge pro-oxidant species [60,61]. The scavenging capacity of olive fruit and leaf extracts enriched with oleuropein against the ROS and RNS studied is summarized in Table 3.

The *O. europaea* fruit and leaf extract enriched with 30% oleuropein (O30) was the most effective scavenger for all ROS and RNS tested. The highest quenching efficiencies were achieved for NO^●^ (IC_50_ = 1.7 µg/mL) and O_2_^●−^ (IC_50_ = 20.1–29 µg/mL). It is noteworthy that a detailed evaluation of the in vitro radical scavenging activity of industrial olive byproduct-enriched extracts is provided for the first time in this paper, comprising an opening field of research. In fact, our values are higher than those reported in commercial micronized powdered olive leaves by Goldschmidt et al. for all ROS/RNS assays [52,62].

O_2_^●−^ is the first radical formed during oxidative processes by NADPH oxidase and is quickly transformed into more potent reactive species [59]. The O_2_^●−^ scavenging efficiency decreased in the following order: gallic acid > O30 extract > O20 extract > catechin. Among samples, the best O_2_^●−^ scavenger was the O30 extract (IC_50_ = 20.1 µg/mL), while the O20 extract displayed the lowest quenching capacity based on the highest IC_50_ value (IC_50_ = 29 µg/mL). Gallic acid (IC_50_ = 6.0 µg/mL) exhibited the highest O_2_^●−^ scavenging ability, while catechin showed the lowest quenching capacity (IC_50_ = 43 µg/mL). Significant differences (*p* < 0.05) were observed for gallic acid and catechin, while O20 and O30 extracts were not significantly different (*p* > 0.05). However, both extracts were statistically different (*p* < 0.05) from the positive controls. These results were in line with those documented for *Psidium cattleianum* pulp extract (IC_50_ = 20.6 µg/mL) [61]. Nevertheless, olive leaves, walnut leaves and hardy kiwi leaves revealed a lower capacity to quench O_2_^●−^ (with IC_50_ values of 0.047–0.386 mg/mL, 47.6 µg/mL and 53.74 µg/mL, respectively) [63,64,65]. Additionally, Kumar et al. also reported lower O_2_^●−^ quenching power for a methanolic extract of *Indigofera cassioides* leaves (IC_50_ = 232.0 µg/mL) [66]. On the other hand, *Castanea sativa* and *Quercus robur* leaf extracts exhibited higher O_2_^●−^ scavenging ability associated with lower IC_50_ values (13.6 and 11.0 µg/mL, respectively) [67].

HOCl is a powerful reactive species which results from the reaction between H_2_O_2_ and chloride ions catalyzed by myeloperoxidase enzymes abundantly present in mammalian granulocytic leukocytes [59]. Concerning to the HOCl scavenging assay, catechin (IC_50_ = 0.42 μg/mL) was the best quencher, followed by gallic acid (IC_50_ = 4.0 μg/mL), O20 (IC_50_ = 33 μg/mL) and O30 extracts (IC_50_ = 34 μg/mL). The extracts presented similar results evidenced by the absence of statistical differences (*p* > 0.05). No significant differences were observed for catechin and gallic acid. Otherwise, both extracts displayed significant differences (*p* < 0.05) towards positive controls. Similar HOCl scavenging capacities were reported for *Citharexylum solanaceum* pulp, skin and seed extracts (IC_50_ = 22.8–64.0 μg/mL), as well as *Caryocar villosum* pulp (IC_50_ = 3.6–299.0 μg/mL) and *P. cattleianum* skin extracts (IC_50_ = 32.0 μg/mL) [60,61,68]. Moreover, the present results are substantially higher than those obtained by Reinoso et al. for *C. sativa* leaves (IC_50_ = 63.8 µg/mL) [69].

NO^●^ is a reactive species generated by the conversion reaction of L-arginine to L-citrulline catalyzed by nitric oxide synthase. This reactive species is involved in various physiological processes, including inflammation and immune response, and may be used as a precursor of peroxynitrite (ONOO^−^), which is a more damaging reactive species [59]. The IC_50_ values of extracts and positive controls in this scavenging assay increased in the following order: gallic acid < catechin < O20 extract < O30 extract, with gallic acid (IC_50_ = 0.20 µg/mL) and catechin (IC_50_ = 0.95 µg/mL) exhibiting the highest NO^●^ quenching activity. The extracts were significantly different (*p* < 0.05) when compared to gallic acid and catechin. Otherwise, no significant differences (*p* > 0.05) were detected between both extracts that exhibited similar IC_50_ values (1.65 and 1.73 µg/mL for O20 and O30 extracts, respectively). These results were in accordance with those reported for walnut leaves (IC_50_ = 1.95 µg/mL) [64]. A lower NO^●^ quenching potential was observed for leaf extracts from *C. sativa* (IC_50_ = 3.10 µg/mL), *Q. robur* (IC_50_ = 3.13 µg/mL) and *A. arguta* (IC_50_ = 3.80 µg/mL) [64,67]. Likewise, when compared to *P. cattleianum* skin and pulp (IC_50_ = 2.2–6.8 µg/mL) and *C. villosum* pulp extracts (IC_50_ = 2.8–142 µg/mL), the obtained results were also considerably higher [60,61]. In addition, Puerta et al. investigated the scavenging power of the main phenolic compounds identified in olive oil, namely, oleuropein, caffeic acid and hydroxytyrosol. Caffeic acid revealed the highest NO^●^ quenching power (65% of inhibition), while the other polyphenols only achieved 50% of inhibition [70].

The promising outcomes of *O. europaea* byproduct extracts in the scavenging of ROS and RNS studied may be due to the phenolic composition, especially oleuropein as reported in the previous sections, whose scavenging capacity has already been described in previous studies [70,71].

#### 3.2.3. Enzyme Inhibition

Concerning the cosmetic potential evaluation of olive byproducts, it is noteworthy that studies in the literature are scarce, although surprisingly, the number of olive-based cosmetic products in the market is very high. Several studies demonstrate that olive byproducts possess antipigmentation potential inhibiting tyrosinase and other cosmetic properties. For example, the *O. europaea* flowers showed interesting elastase and collagenase inhibition potentials [72]. In fact, hyaluronidase and elastase inhibitory activities have been evaluated in other different herbal extracts [73,74]. By catalyzing the hydrolysis of hyaluronic acid, hyaluronidase decreases the viscosity of body fluids and increases the permeability of connective tissues [75]. In the present study, the hyaluronidase inhibition test showed values of IC_50_ = 55 ± 1 μg/mL for O20 and IC_50_ = 100.1 ± 0.8 μg/mL for O30, which are higher than those reported previously for other plant extracts [74]. Interestingly, O20 presented a better result than O30, which could be related to the presence of some iridoids that were only detected in O20. Regarding elastase, this enzyme plays an important role in skin aging since the excessive hydrolysis of the dermal elastin fiber network leads to the loss of skin elasticity and consequent skin sagging [76]. In O20 and O30 extracts, the percentage of inhibiting elastase was 19.08 ± 0.03% and 20.12 ± 0.05% at 250 μg/mL, respectively. It is noteworthy that Puerta et al. investigated the inhibitory effects of the major bioactive compounds present in virgin olive oil on elastase activity [70]. Similar elastase inhibition percentages were reported for oleuropein extracted from olive leaves, hydroxytyrosol and caffeic acid at concentrations of 1 and 0.5 mM (65–75% and 50–55% of inhibition, respectively) [70]. The promising potential of these byproducts against skin damages related to aging by elastase and hyaluronidase inhibition was reported for the first time, providing a starting point of cosmetic development.

#### 3.2.4. Cell Viability

The effects of O20 and O30 extracts on skin cell lines are of utmost importance to evaluate the potentialities of these extracts as active cosmetic ingredients. Keratinocytes are the major cells found in the epidermis, which is the outermost skin layer and where cosmetics are applied [64]. The viability of keratinocytes was investigated as a preliminary study to ascertain the concentrations of extracts that showed absence of toxicity, which is a mandatory requirement for cosmetic ingredients [33,64]. In this way, keratinocytes, as the most superficial skin cells, were exposed to different concentrations (0.1–1000 μg/mL) of both extracts, medium (positive control) and triton X-100 (negative control). The results are summarized in Table 4.

The HaCaT cell line viability did not decrease after exposure to different concentrations of O20 and O30, showing a viability increase of around 100% until a concentration of 100 μg/mL. Until this concentration, no significant differences (*p* > 0.05) were detected between the extracts and medium exposure. Nevertheless, at the highest concentration tested (1000 μg/mL), the cell viability decreased to 61.05 and 42.06%, respectively, after exposure to O20 and O30 extracts. The differences observed between O20 and O30 may be due to the different bioactive compounds extracted, as reported in Table 1. To the best of our knowledge, this is the first study that explores the effects of these commercial extracts on skin cell lines by MTT assay. Nevertheless, Schlupp et al. investigated the viability of keratinocytes by WST-1 assay after exposure to an olive mill wastewater extract [77]. The results revealed a considerable decrease in HaCaT viability for the extract dilution of 1:100 (70.9%) after 72 h. Otherwise, extract dilutions from 1:200 to 1:1000 showed HaCaT viabilities above 100% [77]. The present results are also in line with those obtained by Lameirão et al. for chestnut extracts obtained by ultrasound-assisted extraction [33]. The authors reported viabilities around 65% for keratinocytes after exposure to a concentration of 1000 μg/mL. In another study, Marangi et al. exposed keratinocytes to hardy kiwi leaves extracted by multifrequency multimode modulated technology, reporting a viability of 97% after exposure to a concentration of 500 μg/mL [64]. It is likely that the different compounds extracted are responsible for these different results. In this sense, MTT assay revealed an optimal range of O20 and O30 extract concentrations up to 100 μg/mL, suggesting the safety of olive fruit and leaf extracts for skin application and encouraging their use as cosmetic ingredients. However, further studies will be performed to ensure the safety and efficacy of these extracts, particularly concerning ROS effects on skin cell lines (such as HaCaT cells), skin irritation tests using in vitro validated models (Episkin^®^) and ex vivo skin permeation through Franz diffusion cells.

## 4. Conclusions

The phytochemical composition of industrial olive byproducts makes these bioactive extracts attractive ingredients to be incorporated into cosmetic formulations. The extracts screened in the present study were obtained with a feasible and cost-effective scaling-up process using green solvents and inexpensive, renewable and abundant raw material. However, it is necessary to stimulate innovative approaches in cosmetics and to explain their potentialities to olive producers in order to highlight their use as new raw materials.

In the present study, a robust HPLC-QTOF platform was employed to identify a total of 49 compounds; among them, 28 were found in both O20 and O30 extracts. A higher abundance of oleuropein and its related compounds was found in O30, whereas O20 was characterized by a major number of iridoids. Moreover, this work reported for the first time in olive leaves the presence of oleuropein-related compound (**48**), iridoids (**12**, **15**, **16**, **17**, **21**, **25**), paniculin (**26**) and other compounds (**7** and **24**). All detected compounds were demonstrated to possess a strong potential against oxidative stress and aging as reflected in bioactivity performed assays. Specifically, O30 showed higher values in all experiments, except in HOCl scavenging and hyaluronidase inhibition, where O20 obtained the best results. Regarding cell viability assays, between 0.1 and 100 µg/mL, O20 and O30 did not lead to a decrease in viability. However, at the highest concentration tested (1000 µg/mL), both extracts resulted in viabilities of around 42 and 62%, respectively.

The promising potential of industrial olive byproducts as cosmetic ingredients demonstrated in this work is a great starting point for future perspectives that can be concluded with these industrial oleuropein-rich extracts from olive byproducts as bioactive ingredients, allowing the development of new added value products under a biocircular economy model.

## Figures and Tables

**Table 1:** Chemical characterization of bioactive compounds detected in olive byproduct extracts by HPLC-QTOF.

Compound Number	Proposed Compound	RT	*m/z*	Molecular Formula	MS/MS	O20	O30
**1**	Gluconic acid	3.8	195	C6H12O7	129	X	X
**2**	Sucrose	4.98	341	C12H22O11	179	X	X
**3**	Citric acid	6.49	191	C6H8O7	111	X	
**4**	Vanillin	6.63	151	C8H8O3	-		X
**5**	Methyl xylobioside	7.19	295	C11H20O9	181, 151, 191	X	
**6**	Methyl gallate glucoside	7.51	563	C14H18O10	277	X	
**7**	Leonuriside	8.14	331	C14H20O9	169, 139	X	
**8**	Methyl xylobioside	8.65	295	C11H20O9	153	X	
**9**	Oleoside Isomer 1	8.88	389	C16H22O11	137, 295	X	X
**10**	Loganic acid	9.32	375	C16H24O10	315, 213, 209	X	X
**11**	Oleoside Isomer 2	10.11	389	C16H22O11	183, 121	X	X
**12**	Aralidioside	10.59	447	C18H24O13	153	X	X
**13**	Hydroxytyrosol	11.06	153	C8H10O3	123, 135	X	X
**14**	Taxifolin	11.84	303	C15H12O7	161, 179, 153	X	
**15**	Iridoid glicoside derivative	12.26	553	C22H34O16	181, 411	X	
**16**	Allobetonicoside	12.95	505	C21H30O14	161	X	
**17**	Eriobioside	13.23	567	C23H36O16	181, 223, 161, 341, 403, 505	X	
**18**	Elenolic acid glucoside Isomer 1	13.64	403	C17H24O11	161	X	X
**19**	Elenolic acid glucoside Isomer 2	14.04	403	C17H24O11	161	X	
**20**	Loganin	15.65	389	C17H26O10	327, 267	X	
**21**	Acetylbarlerin	16.25	489	C21H30O13	145, 163, 327	X	X
**22**	Oleoside Isomer 3	17.46	389	C16H22O11	345	X	X
**23**	Benzyl primeveroside	17.98	401	C18H26O10	223	X	X
**24**	Cinnamoside	18.21	517	C24H38O12	387, 459, 409, 175	X	X
**25**	Depressine	19.45	687	C25H30O13	525, 161		X
**26**	Paniculatin	20.06	593	C27H30O15	353, 383, 473, 175		X
**27**	Kaempferol diglucoside	21.77	609	C27H30O16	447, 285, 197, 153	X	X
**28**	Phenethyl primeveroside Isomer 1	23.41	415	C19H28O10	151, 175, 223	X	
**29**	Hydroxyoleuropein Isomer 1	23.78	555	C25H32O14	151		X
**30**	Phenethyl primeveroside Isomer 2	23.87	415	C19H28O10	151, 123	X	X
**31**	Oleuropein glucoside Isomer 1	25.35	701	C31H42O18	315, 285, 447, 337	X	X
**32**	Verbascoside	26.1	623	C29H36O15	161, 461	X	X
**33**	Syringaresinol	26.87	417	C22H26O8	181, 166, 387		X
**34**	Hydroxyoleuropein Isomer 2	26.93	555	C25H32O14	161, 417, 181	X	X
**35**	Calceolarioside A	27.2	477	C23H26O11	161		X
**36**	Kaempferol rutinoside	27.88	593	C27H30O15	285	X	X
**37**	Luteolin glucoside	28.2	447	C21H20O11	285	X	X
**38**	Oleuropein glucoside Isomer 2	29.31	701	C31H42O18	609, 300, 539, 269	X	X
**39**	Methoxyoleuropein	29.74	569	C26H34O14	151, 223, 537, 403, 553	X	X
**40**	Oleuropein Isomer 1	30.36	539	C25H32O13	307, 275, 149, 377	X	X
**41**	Luteolin glucoside	31.85	447	C21H20O11	285	X	X
**42**	Oleuropein Isomer 2	32.38	539	C25H32O13	307, 275, 403, 149, 377	X	X
**43**	Ligstroside	32.98	523	C25H32O12	291, 259, 361	X	X
**44**	Oleuropein Isomer 3	33.4	539	C25H32O13	307, 275, 121, 223	X	X
**45**	Oleuropein Isomer 4	34.45	539	C25H32O13	307, 275, 153, 377	X	X
**46**	Oleoeuropein aglycone Isomer 1	35.16	377	C19H22O8	307, 149, 275	X	X
**47**	Oleoeuropein aglycone Isomer 2	35.79	377	C19H22O8	307, 149, 139, 11, 275		X
**48**	Oleuropein derivative	36.53	763	C36H44O18	539, 307		X
**49**	Oleoeuropein aglycone Isomer 3	37.07	377	C19H22O8	307, 275		X

MS/MS: tandem mass spectrometry.

**Table 2:** Total phenolic content and antioxidant capacity of both olive byproduct extracts.

	TPC ^a^	FRAP ^b^	TEAC ^c^	ORAC ^c^
**O20**	193 ± 9	1.66 ± 0.03	0.80 ± 0.05	3.91 ± 0.01
**O30**	217 ± 3	1.90 ± 0.06	0.95 ± 0.02	3.99 ± 0.08

^a^ mg GAE/g dry extract, ^b^ mmol eq. FeSO_4_/g dry extract, ^c^ mmol eq. Trolox/g dry extract. Values are expressed as mean ± standard deviation.

**Table 3:** ROS/RNS of both olive byproduct extracts (IC_50_, μg/mL).

	HOCl	O2^●−^	NO^●^
***O. europaea byproduct extracts***			
**O20**	33 ± 2 ^a^	29 ± 2 ^a^	1.7 ± 0.1 ^a^
**O30**	34 ± 3 ^a^	20.0 ± 0.6 ^a^	1.7 ± 0.1 ^a^
***Positive controls***			
**Gallic acid**	4.0 ± 0.4 ^b^	6.0 ± 0.5 ^b^	0.20 ± 0.03 ^b^
**Catechin**	0.42 ± 0.03 ^b^	43 ± 4 ^c^	0.95 ± 0.04 ^c^

IC_50_ = In vitro concentration required to reduce the reactivity of the tested reactive species by 50% (mean ± standard error of mean). Different letters (^a^, ^b^, ^c^) in the same column indicate significant differences between extracts and positive controls (*p* < 0.05).

**Table 4:** Effects of exposure to O20 and O30 extracts on the viability of keratinocytes at different concentrations, as measured by the MTT assay.

Concentrations (μg/mL)					
	0.1	1	10	100	1000
**Medium**	100.01 ± 16.05	100.01 ± 16.05	100.01 ± 16.05	100.01 ± 16.05	100.01 ± 16.05
**Triton X-100**	0.00 ± 0.00	0.00 ± 0.00	0.00 ± 0.00	0.00 ± 0.00	0.00 ± 0.00
**O20**	100.23 ± 20.82	100.21 ± 21.94	113.60 ± 22.86	106.79 ± 17.60	61.05 ± 10.86 *
**O30**	100.63 ± 7.08	105.65 ± 14.45	98.92 ± 14.82	107.31 ± 8.64	42.06 ± 5.33 *

Values are expressed as mean ± standard deviation (*n* = 4). * *p* < 0.05 vs. control. MTT: 3-(4,5-dimethylthiazol-2-yl)-2,5-diphenyltetrazolium bromide.

## Data Availability

Not applicable.
